# Electronic patient record use during ward rounds: a qualitative study of interaction between medical staff

**DOI:** 10.1186/cc7134

**Published:** 2008-11-24

**Authors:** Cecily Morrison, Matthew Jones, Alan Blackwell, Alain Vuylsteke

**Affiliations:** 1 Computer Laboratory, University of Cambridge, 15 JJ Thompson Avenue, Cambridge, CB3 0FD, UK; 2 Judge Business School, University of Cambridge, Trumpington Street, Cambridge, CB2 1AG, UK; 3 Papworth Hospital, NHS Foundation Trust, Cambridge, CB23 3RE, UK

## Abstract

**Introduction:**

Electronic patient records are becoming more common in critical care. As their design and implementation are optimized for single users rather than for groups, we aimed to understand the differences in interaction between members of a multidisciplinary team during ward rounds using an electronic, as opposed to paper, patient medical record.

**Methods:**

A qualitative study of morning ward rounds of an intensive care unit that triangulates data from video-based interaction analysis, observation, and interviews.

**Results:**

Our analysis demonstrates several difficulties the ward round team faced when interacting with each other using the electronic record compared with the paper one. The physical setup of the technology may impede the consultant's ability to lead the ward round and may prevent other clinical staff from contributing to discussions.

**Conclusions:**

We discuss technical and social solutions for minimizing the impact of introducing an electronic patient record, emphasizing the need to balance both. We note that awareness of the effects of technology can enable ward-round teams to adapt their formations and information sources to facilitate multidisciplinary communication during the ward round.

## Introduction

Electronic patient records (EPRs) are progressively being implemented in many hospitals. Although there is a growing literature addressing the difficulties of EPR implementation – including doctor usage [1], user response to implementation [2], doctor–patient communication [3], and organizational issues [4] – it is a multifaceted issue with much still to be understood [5]. In fact, most previous research provides useful guidelines for various aspects of implementation but the need remains to '[overcome] the cognitive and behavioural barriers of machine-man interactions' in order to reap the promises of EPR systems [5].

Ward rounds are a notable hospital context in which staff work as a group. Technology designed for a single user, like most EPR systems, poses challenges to group interaction – an issue not widely discussed in the healthcare literature. Using theory from the field of human–computer interaction, we evaluate EPR usage through a comparative study of interaction during ward rounds in an intensive care unit (ICU) that transitioned from a paper patient record to an EPR. We highlight the role of physical group formation and the ergonomics of each system in facilitating or hindering group use of patient records.

## Materials and methods

### Background

The medical lead of the ICU described in the present paper initiated a switch to an EPR from a paper record in order to improve record keeping such as prescription legibility, adherence to guidelines, and research and development opportunities. Funding was approved in spring 2006 for the purchase of a commercially available clinical information system (Metavision; iMDsoft; Needham; Massachusetts; USA), which was deployed bed by bed across the unit on 6 November. Between the funding approval and the deployment, an implementation steering group established a plan to introduce the system, overseeing the customization process to meet the needs of the unit as well as examining probable changes, or disruptions, to work practices.

The implementation steering group was particularly concerned about how the new system might impact job satisfaction and communication between various medical practitioners. With the agreement of the trust authorities and the Caldicott guardian, the steering group invited a multidisciplinary team of researchers including social and computer scientists to observe and record working practices pre and post change, starting in summer 2006. Their observations were fed back to the implementation steering group on a regular basis to help them ensure a smooth migration from paper patient records to EPRs. The researchers were not funded by the hospital, and decisions to adjust work practices lay entirely with the implementation steering group.

As the research does not contain any patient data or interaction, it has been classified as an audit by the Cambridgeshire Research Ethics Committee and therefore does not require ethics approval. All of the medical practitioners observed or interviewed, however, were aware of the purpose of the studies – particularly the consultant videoed (author AV).

### Data analysis methodology

Our analysis aims to answer the following question: How does interaction during clinical ward rounds vary when an EPR is used in place of a paper record?

Given the complex nature of interaction of multidisciplinary communication in an ICU, we have chosen to triangulate three types of qualitative data: video-based interaction analysis, observation, and interviews. Video-based interaction analysis is a technique intended 'to identify regularities in the ways in which participants utilize the resources of the complex social and material world of actors and objects' [6]. It is a technique particularly useful for observing, and perhaps understanding, the impetus of subtle changes in behaviour, and is the main source of data presented in this paper. Observation provides background information for the video analysis, and was used to ensure the analysis was not limited by the scope of the camera's lens. Interviews are useful for gathering information on how the system is used, and in this case provided validation of hypotheses generated during video analysis about the interaction. Quantitative measures were not used as it was unlikely they would provide external validity in this situation of complex social interaction between specialized participants [7].

The primary function of ward rounds is to provide an occasion for the medical team to review and integrate information as a group in order to make a clinical decision [8-10]. As the paper and electronic records present and allow access to information in different ways, the change of record is likely to affect interaction. We therefore chose to compare how interaction was achieved with each type of record. The ward-round discussion needs to ensure that all necessary information is presented but time is not wasted. The interaction, then, is a negotiation of how the topic of conversation advances and of how people can enter the conversation [11]. Kendon demonstrates in his theory F-formation Systems that groups negotiate interaction (often unconsciously) by adapting group formation, body orientation, and posture [12].

Using this analytical perspective to support the video analysis, along with the data from observation and interviews, we demonstrate how the ergonomics of the two record types affect group formation. We consequently demonstrate the way in which members of the ward round team use body orientation and posture to negotiate interaction in terms of conversation advancement and entry.

### Data acquisition

Ward rounds were video recorded by author CM, trained in anthropological techniques of field observation and video-based interaction analysis. Video recordings were obtained at three points during the observation period of 13 months: 1 month prior to deployment of the EPR, 4 months after deployment of the EPR, and 1 year after deployment of the EPR. Each time, six separate, randomly selected patient discussions were filmed. To enable comparison, those ward rounds selected for filming were always managed by the same consultant.

Images from the video recordings were shared with members of the implementation steering group – including the consultant videoed – 6 months after deployment of the system, and the effects of the introduction of the EPR on group interaction were discussed. Patient privacy was ensured at all times by avoiding capture of images that might allow patient identification.

Video footage was complemented by observation during the above three periods both at the time of filming and on another day. Further observation took place the week after deployment and of other consultants throughout the observation period. Three rounds of interviews were conducted at similar time intervals by author MJ. Seven participants were drawn from all medical and nursing roles, including at least one teaching nurse who was responsible for carrying out the training on the system. Effort was made to interview the same people each time, but due to scheduling there were some substitutions.

### Setting

#### Intensive care unit

The ICU, consisting of 25 critical care beds, is part of a specialist hospital that concentrates on all aspects of adult cardiothoracic care. Approximately 70% of admissions are patients recovering from cardiac surgery. The unit has a high turnover with a 3-day median duration of stay.

There are approximately 200 practitioners working in the ICU, with at least 30 on duty at any one time. These practitioners include a consultant intensivist (senior doctor), two specialist registrars on duty for critical care (junior doctors), one sister in charge of the nurses in the unit (head nurse), one senior nurse in charge of each of three clusters of patient beds, one nurse looking after each of the 25 patients, the intensive care pharmacist, the intensive care dietician, and a team of physiotherapists. The large nature of the unit results in the on-duty group changing configuration regularly.

#### Multidisciplinary ward round

The ward-round team is made up of a member from each of the above roles as appropriate (for example, the bed nurse for that patient) – consultant, two registrars, head nurse, senior nurse, bed nurse, pharmacist, dietician and head of physiotherapy – comprising eight to 10 people, with possible additional medical students or support from consultants, microbiologists, or surgeons. Although the team structure is consistent, different individuals may fulfil each role on a given day.

The multidisciplinary ward-round team travels from bed to bed each morning to review patient progress. The team updates itself on each patient's condition through discussion and chart review, and decides upon the patient's plan for the day. As the round is business orientated, aiming to review all 25 beds in the short period of time ahead of postoperative admissions, little time is devoted to teaching. The daily plan and prescriptions, however, are filled out during the ward round when possible.

The ward round begins with one of the registrars presenting the most pertinent details of the patient and any recent changes. The discussion that ensues is led by the consultant working systematically through a number of issues, as appropriate. Any member of the ward-round team can contribute to discussion or may be specifically called on by the consultant for their expertise. The ward round is usually close by the consultant asking 'is there anything else?' Although there is no particular structure for participation by the medical staff, the consultant videoed (author AV) strongly encourages participation from all of those involved in the ward round.

### Patient records

#### Paper record

**Figure 1:**
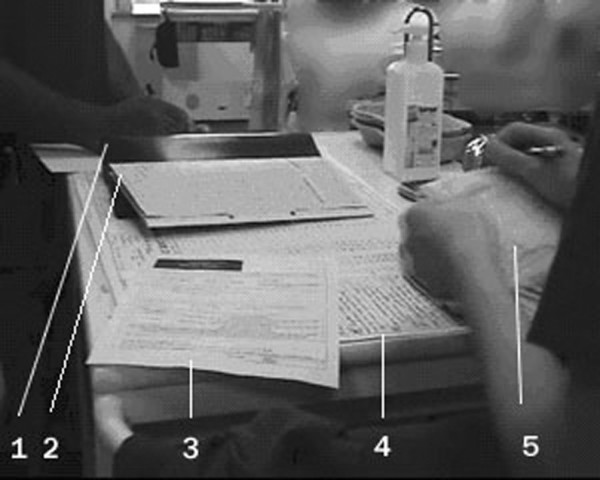
**Paper patient medical record**. 1, binder; 2, drug chart; 3, patient plan of the day; 4, observation chart; 5, personal notes.

The paper patient record, shown in Figure 1, consisted of three specific types of form (the observation chart, the drug chart, and the plan of the day) and a folder or binder for miscellaneous and patient-specific forms and papers. The observation chart was A3-size paper that lay flat on the nurse's table. The nurse plotted vital signs on it regularly, recorded blood test results, wrote other medical notes, and kept nonmedical care information on the reverse side. A new chart was used each day and was placed on top of the old one.

#### Electronic patient record

**Figure 2:**
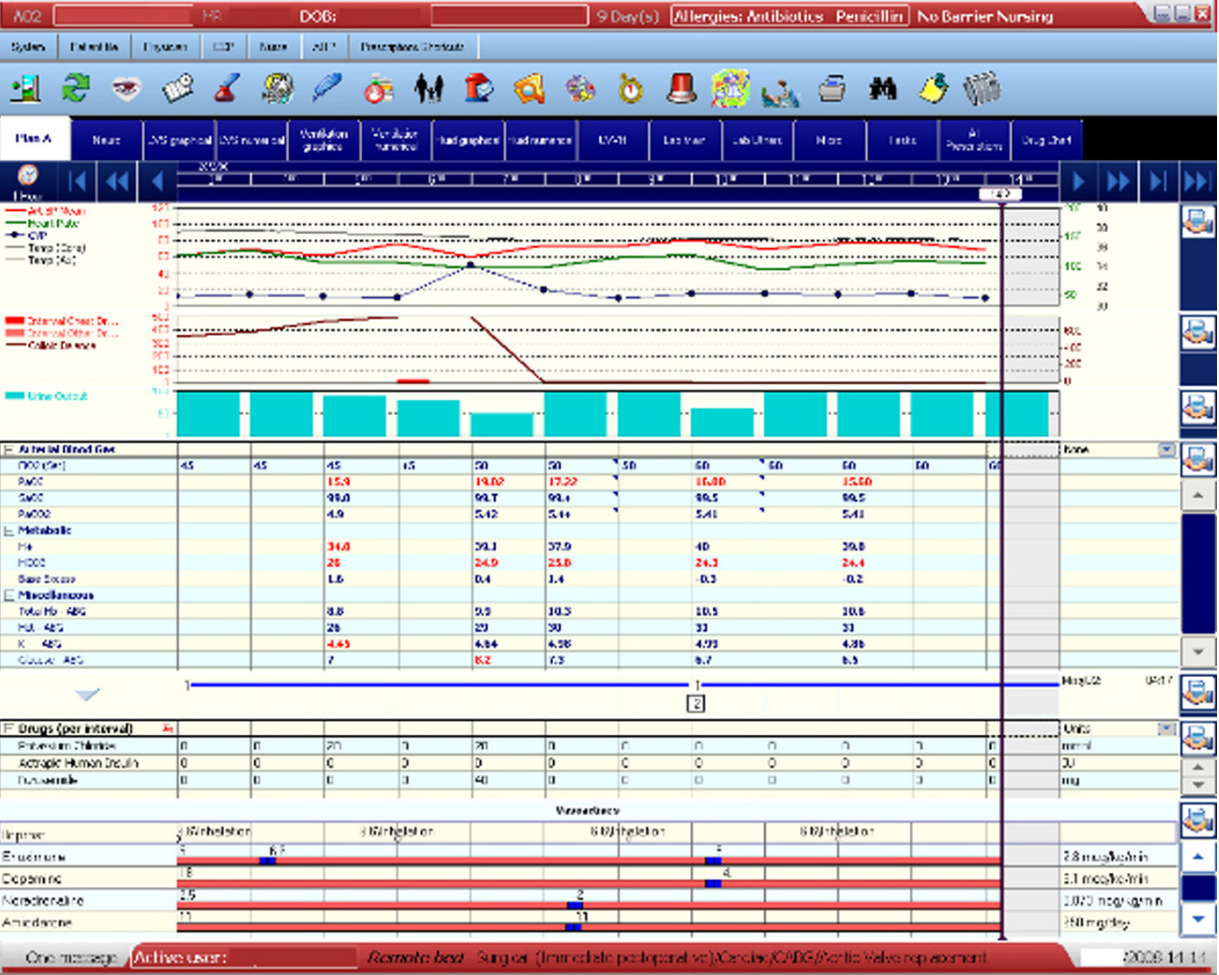
**Electronic patient record summary screen**. The summary screen, displayed on a 19-inchscreen at the patient bedside, contains the most important information about the patient's condition; it is the primary screen used during the ward round. All other screens – that is, those giving detailed data on particular aspects of a patient's condition and treatment – are accessible via tabs displayed across the top of the summary screen. Patient details have been removed.

The EPR, provided by Metavision, is a system developed specifically for intensive care use, allowing full integration of data gathered at the bedside into a highly customizable interface. The record includes parameters from ventilators, monitoring devices, laboratory results, prescriptions, and medical and nursing records. A summary screen that displays the most important information about the patient's condition was developed for use during the ward round. All other screens – that is, those giving detailed data on particular aspects of a patient's condition and treatment – are accessible via tabs displayed across the top of the summary screen as shown in Figure 2.

A multidisciplinary team at the hospital designed the initial interface to be used in the unit before implementation. The software allows the clinical design team to make changes on the fly and to react to staff feedback, such that the interface is constantly evolving. Consequently, there were no major software issues and, fortunately, no technical difficulties.

The EPRs are displayed on 19-inch monitors positioned on an adjustable height trolley at the end of each bed. The trolley can be moved around the bed, but its range is limited because of the wire connections to the ceiling. The screen cannot be rotated, but the trolley itself can. The trolley is generally not moved during the ward round, although the bed nurses frequently adjust the trolley for themselves.

## Results

### Group formation

**Figure 3:**
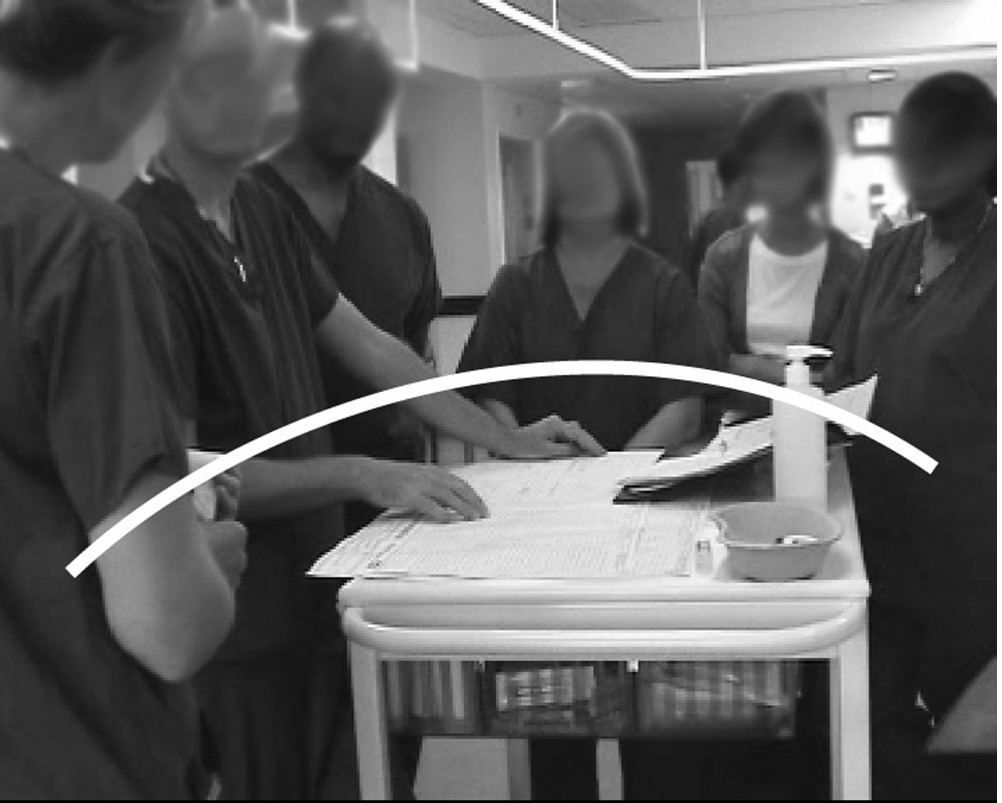
**The ward-round team using the paper patient record**. Classical distribution observed when the paper record was in use; the medical team distributed itself in a horseshoe shape around a table at the end of the bed, with the consultant at the top.

**Figure 4:**
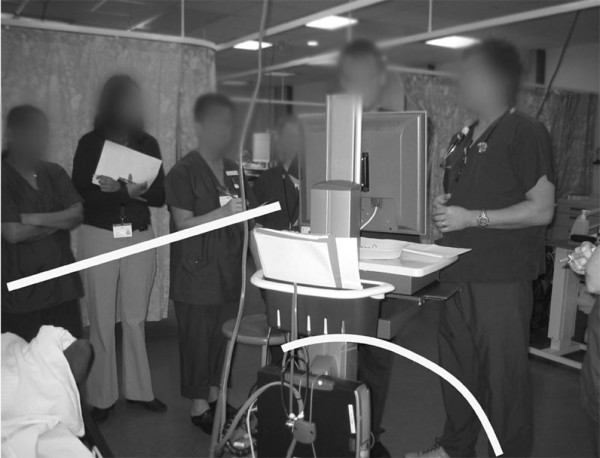
**The ward-round team using the electronic patient record 4 months after implementation**. A new formation, consistently observed during the first few months after implementation of the electronic patient record, with two rings of people. In the first week of implementation, the group attempted to form a single ring around the computer – but this proved impractical as no one could see the screen, so the double ring was taken up.

**Figure 5:**
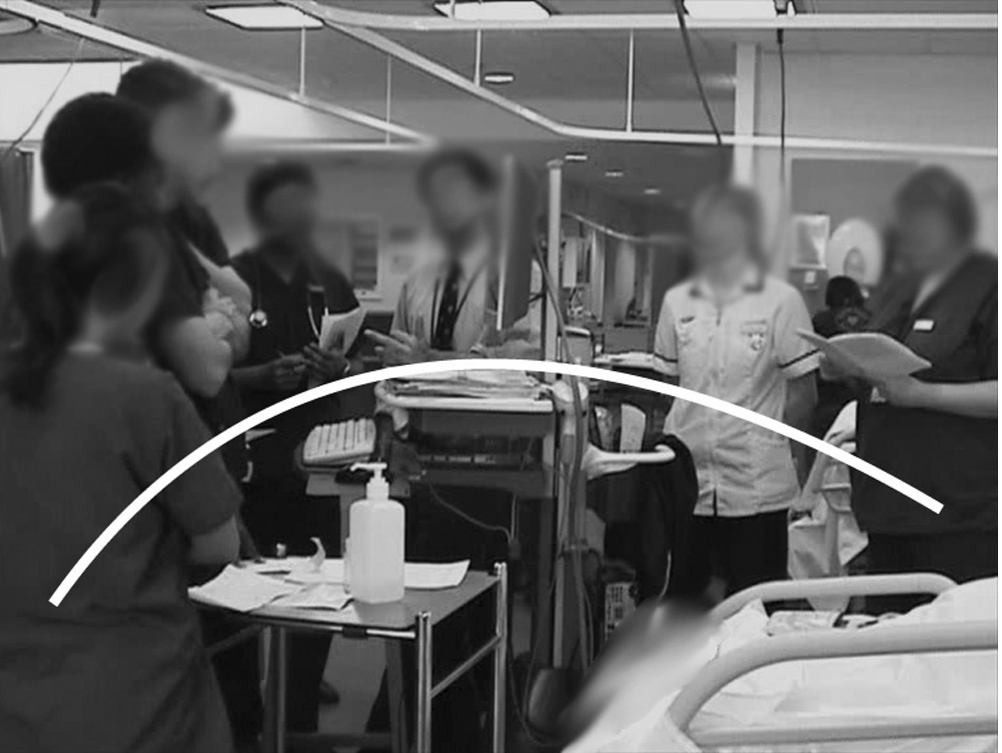
**The ward-round team using the electronic patient record 1 year after implementation**. The same practitioner group 1 year later. The group once again has formed a single ring, this time around the patient. The medical staff looked at and touched the patient significantly more. The consultant stood further back from the display, keeping more of the group in his peripheral vision.

Group formation during the multidisciplinary ward round changed considerably during the observation period, as demonstrated in Figures 3, 4, and 5.

### How is the conversation advanced?

#### Body orientation

One month prior to deployment of the EPR, with the paper record the consultant took his position at the head of the table, leaning in towards the paper and spreading his hands across it. His body orientation towards the charts suggests them to be the primary focus of the conversation and everyone else orients towards the charts as well.

Four months after deployment of the EPR, the consultant's orientation towards the screen displaying the electronic record does little to direct the attention of those who cannot see the screen. The consultant in this case loses his ability to guide the focus of the group and, not surprisingly, the gaze of the medical staff in the outer ring tends to wander.

One year after deployment of the EPR, while the consultant remains oriented toward the computer screen, the registrars and medical staff have adjusted to form a horseshoe around the patient's bed. From this position, the ward-round team can easily monitor the consultant's gaze and reactions toward the conversation. The team frequently follow his gaze to the patient and monitor or keep their attention on the faces of those speaking. The consultant leads the conversation, not by focusing the team's attention on the data but on the conversation itself.

#### Posture

One month prior to deployment of the EPR, the posture taken by the consultant – as shown in Figure 3, with his hand spread across the paper charts – indicates his control of the conversation's progression. From this position, he is able to point at information, guiding the attention of the group and often the people speaking. Numerous instances were observed of the registrars modifying their presentations to match the data being pointed at. The consultant further regulated the content of the discussion by pulling particular charts into the middle of the table, thus switching the topic of conversation.

Four months after deployment, when using the EPR, the person with the mouse – typically the consultant – had the same ability to guide the conversation noted previously. He could only, however, direct the focus of those who could see the screen.

One year after deployment of the EPR, the upright posture of the consultant and his position, slightly farther back from the computer so that his face could be seen easily, facilitated the ward-round team in following the consultant's focus. It also allowed the consultant to monitor the attention of the ward-round team. A question to one of the medical staff was used twice to refocus that person, providing another tool for the consultant to lead the team.

### How can people enter the conversation?

#### Body orientation

One month prior to deployment of the EPR, within the ward round, side conversations often took place between the nurses or between the pharmacist and another medical staff member. To request such a conversation when using the paper record, a chart was picked up and both parties reoriented themselves to it. In this position, the parties could have a conversation while still visually monitoring the main conversation.

Four months after deployment, the EPR offered no means to invite reorientation; neither could the main conversation be monitored visually as the only connection between the rings in the formation was aural.

One year after deployment of the EPR, side conversations remained rare and were limited to a sentence or two, with the two parties occasionally shifting closer to one another but not reorienting themselves. Following the completion of the ward round, however, there would be numerous small conversations. As the intensive care pharmacist commented, before the introduction of the EPR she would have reviewed and made changes to drug charts during the ward round, now she focused on the team discussion during the round and made her interventions afterwards.

#### Posture

One month prior to deployment of the EPR, posture was a significant indicator of participation in the conversation. Leaning into the circle provided a clear indication of one's desire to speak, and this was usually granted by the consultant reorienting towards that person. Another way of starting the conversation was to place a chart in front of the consultant or registrar, putting the onus on one of them to open the discussion. Direct verbal requests to enter the conversation were either ignored or treated tersely.

Four months after deployment of the EPR, the only means available to medical staff to request entry into the conversation was through direct verbal interruption. Not surprisingly, there was a decrease in communication between doctors and nursing staff.

One year after deployment of the EPR, there were two ways through which people entered the conversation. Either a member of the team stepped into the horseshoe to gain attention, or the consultant logged out of the EPR, stood back from the computer, and asked whether there was anything else to discuss. This formation allows the consultant to see everyone, and results in a greater likelihood that these requests will be acknowledged. General questions from the consultant to the team also gave medical staff a reason to speak up, with staff often leaning into the formation to answer the question or point to something.

The almost circular formation and a less constant orientation towards the data seemed to change the dynamic of the interaction. Medical staff responded to discussions more frequently without necessarily requesting the focus of the group. Their confidence to speak out may also have been bolstered by greater preparation before the ward round – a phenomenon several staff reported as a solution to not being able to see the screen. As with the pharmacist's interventions, therefore, introduction of the EPR meant that certain activities were carried out in series rather than in parallel.

## Discussion

### ICU multidisciplinary communication

Strong multidisciplinary collaboration in an ICU context is known to be beneficial for patient outcomes [13], but is also difficult to achieve [14]. Our results suggest that the physical setup of the EPR, by giving unequal access to the patient's data as well as the consultant's reaction to the data, can lead to decreased interaction or openness of discussion, which may result in the medical staff having less understanding of their patient care goals [15]. Furthermore, the easy access to information that the EPR provides does not encourage the usual trading of information that stimulates multidisciplinary interaction [16] and provides important contextual information not necessarily contained in the EPR. The adjustments seen after the ward-round team became aware of the lack of interaction when using the EPR – a change of formation to allow focus on the conversation rather than the data, as described above, and the use of paper to provide relevant data – are not surprising in that they address the issues of access to data and a need to stimulate multidisciplinary interaction highlighted above.

### Solutions

#### Technology solutions

A number of technical solutions that might ease the blocks to interaction caused by the physical setup were explored. The implementation steering group had discussed sitting ward rounds, in which the EPR is projected onto the wall, but rejected them because they did not include the bed nurse or the patient. Larger screens were considered, but the cost was prohibitive.

The computer science researchers investigated handheld devices, or PDAs, as a way of allowing the ward-round team to change their formations. Preliminary results, however, suggest that this is not as helpful as expected [17]. Handheld devices, like the original display, encourage team members to focus on the information rather than on the interaction, making it difficult to monitor the actions of others and discouraging communication.

Ironically, the one type of technology that was found to be useful was paper printouts containing basic information for each member of the medical staff, which helped them orient towards the interaction. Although EPRs have many benefits, they often do not make a unit paperless. This is a finding common across sectors [18].

#### Social solutions

Often there is not a single technical solution to support complex social environments, but rather a need to balance the technology and the social context to enable existing interaction mechanisms. A first indicator that the technological setup is not facilitating interaction is a broken (noncontinuous) formation. When training time is available, we have, in another article, proposed exercises that ward-round teams can do to better understand how their formations around technology affect interaction by constraining formation in unusual ways and then encouraging the team to discuss possible useful changes in the technology or the interaction [19]. In cases where training is limited, we suggest that the leaders focus on achieving a conversation, on being wary that formation affects interaction and that the substantial amount of information in the EPR might distract rather than add to the interaction, and on encouraging medical staff to adjust as necessary (for example, bringing notes/papers to the ward round).

## Conclusion

The introduction of an EPR into the ICU of the hospital disrupted the way in which the multidisciplinary team organized itself at the patient's bedside, decreasing both the consultant's ability to lead through directing the focus of the group and the opportunity of medical staff to participate in the conversation. Awareness of these disruptions provided by the observing research team and discussions of formations around two records assisted the ward-round team in adapting their behaviour to promote more effective interaction. This adaptation can be seen by an increase in doctor–nurse interaction during the ward round and a decrease in wandering attention seen 1 year after implementation and 6 months after the researchers' findings were discussed with the implementation steering group.

## Key messages

• EPRs are designed for a single user but are frequently used by groups during the ward round.

• Group formation, and the resulting nonverbal behaviour that it allows, is an important way of negotiating who speaks and what is spoken about during ward rounds but can be affected by the ergonomics of the technology used.

• In the example presented, the head consultant loses his ability to direct the conversation and other medical practitioners have difficulty participating in the ward round when using the EPR.

• Prior research into multidisciplinary communication in intensive care suggests that these changes can significantly impact the effectiveness of the interaction.

• We suggest the solution may not be entirely technical, but rather a balance between finding the correct technology and adjusting interaction patterns around it, paying particular attention to formation and access to information.

## Abbreviations

EPR: electronic patient record; ICU: intensive care unit.

## Competing interests

The software developer (iMDsoft; Needham; Massachusetts; USA) and the software UK distributor (Fukuda-Denshi UK; Old Woking; Surrey; UK) have contributed a nonrestricted educational grant to AV's research funds. All other authors declare that they have no competing interests.

## Authors' contributions

CM videoed and observed the ward rounds. MJ conducted interviews and analysed the results. AB and AV contributed to the study design. CM, MJ, and AB carried out the video analysis. CM, MJ, and AV drafted the paper.
